# A bioanalytical UHPLC based method used for the quantification of Thymoquinone-loaded-PLGA-nanoparticles in the treatment of epilepsy

**DOI:** 10.1186/s13065-020-0664-x

**Published:** 2020-02-14

**Authors:** Niyaz Ahmad, Rizwan Ahmad, Sadiq Al Qatifi, Mahdi Alessa, Hassan Al Hajji, Md Sarafroz

**Affiliations:** 1 Department of Pharmaceutics, College of Clinical Pharmacy, Imam Abdulrahman Bin Faisal University, Dammam, Kingdom of Saudi Arabia; 2 Department of Pharmaceutical Chemistry, College of Clinical Pharmacy, Imam Abdulrahman Bin Faisal University, Dammam, Kingdom of Saudi Arabia; 3 Department of Natural Products and Alternative Medicine, College of Clinical Pharmacy, Imam Abdulrahman Bin Faisal University, Dammam, Kingdom of Saudi Arabia

**Keywords:** Thymoquinone, PLGA-nanoparticles, Epilepsy, UHPLC-PDA, Brain bioavailability and pharmacokinetic

## Abstract

To formulate a nanoformulation (PLGA-NPs) and to improve brain bioavailability for thymoquinone (THQ) through intranasal (i.n.) drug delivery, using a newly UHPLC-PDA developed the method and validated. Five different THQ-PLGA-NPs (THQ-N1 to THQ-N5) were prepared by emulsion solvent evaporation method. A new UHPLC method developed and validated for biodistribution studies in the rat’s brain, lungs and plasma. Optimized-THQ-N1-NPs showed a particle size of 97.36 ± 2.01 nm with a low PDI value of 0.263 ± 0.004, ZP of − 17.98 ± 1.09, EE of 82.49 ± 2.38% and DL of 5.09 ± 0.13%. THQ-N1-NPs showed sustained release pattern via in vitro release profile. A bioanalytical method was developed by UHPLC-PDA and validated for the evaluation of pharmacokinetics parameters, biodistribution studies, brain drug-targeting potential (89.89 ± 9.38%), and brain-targeting efficiency (8075.00 ± 113.05%) studies through intranasal administration which showed an improved THQ-brain- bioavailability, compared to i.v. Moreover, THQ-PLGA-NPs improved the seizure threshold treatment i.e. epilepsy increasing current electroshock (ICES) rodent models induced seizures in rats. A significant role of THQ-PLGA-NPs with high brain targeting efficiency of the nanoformulations was established. The reported data supports the treatment of epilepsy.
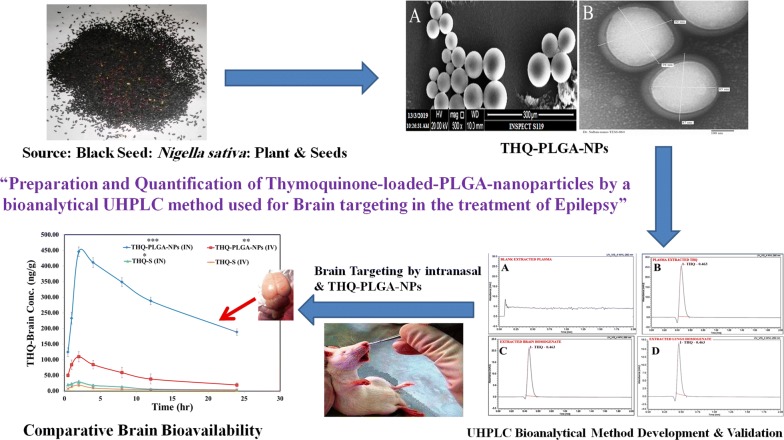

## Introduction

Now a days, peoples (0.5–1%) are suffering from serious neurological disorders in which epilepsy is one of the example [1–3]. A number of treatment approaches (surgery, drugs, and yoga) have been adopted to control this condition. Medicines remain an integral part of part of treatment for seizures [4]. The researchers are focusing on glutamatergic neurotransmission and GABAergic (gamma-aminobutyric acid-ergic) which is the main path of central nervous system for excitatory and inhibitory activity [5, 6]. NHE (sodium-hydrogen exchangers) is also involved in the regulation of seizures activity for the neuronal cells [7, 8].

Medicine from natural sources is one the most important source of treatment as well as a use for the purpose of medicinal, culinary, dietary, and curative. Traditional and alternative source of medicines from plant source rely for development of good health and very less side effects [9]. *Nigella sativa* (Black seed) contain main bioactive active constituent of Thymoquinone (THQ) i.e. Ranunculaceae family, grows commonly in Eastern Europe, Middle East, Western Asia, and Mediterranean countries [10–14]. *Nigella sativa* seeds are traditionally used for the treatment of fever, cough, bronchitis, asthma, influenza, rheumatism, and headache in the Middle East regions, India, Pakistan, and Northern Africa [15–17]. Moreover, in reported preclinical studies it has shown gastro-protective, antitussive, antinociceptive, anti-inflammatory, antihistaminic, anthelmintic, antibacterial, immunomodulatory, antioxidant, hepatoprotective, anticancer, antidiabetic, cardioprotective, protective effects against the ototoxicity, and nephroprotective effects [9]. There are so many benefits for THQ and *N. sativa* with significance to several neurological illnesses. For example, they contain potential effects for anxiolytic, anticonvulsant, antipsychotic, and antidepressant. THQ and *N. sativa* were also used for memory impairments and improve spontaneous functioning as well as reduction of drug tolerance and dependence [9]. Furthermore, neuropharmacological properties of THQ were recently reported the roles of antioxidative and anti-inflammatory in the developed neurological models [18–20].

Now a day, nose-to-brain drug delivery system is an attractive tool for many researchers because of various properties i.e. to bypass the blood brain barrier (BBB), hepatic first pass metabolism as well as a non-invasive route with ease of application [21–23]. It should be known that intranasal formulation should have the characteristics of small residence time of drug in the nasal mucosa/tissue. Mucoadhesive character with high viscosity of any formulation will improve the nasal residence time. Any dosage form designed in such way that has a characteristic of mucoadhesiveness can be enhancing the maximum absorption of drug molecule. Therefore, it is very important to design a dosage form which is more useful for improve nasal mucoadhesive time. Biocompatible and biodegradable nanoparticles are an attractive substitute for nasal gel with in situ formulations for the controlled (alongwith sustained) release of THQ [24–28]. Polymeric nanoparticles are attaining a great attention due to control rate of drug release; prolong duration of therapeutic effect, consuming maximum capability of drug loading (DL), fasten the release of drug due to more surface area, compared to other carriers and drug delivery of drug molecule targeted to the body sites. Polymeric-nanoparticles have so many advantages when compared to intra nasal drug delivery to brain for other drugs: NPs protect drug molecules which are encapsulated in the core of formulation. It is also avoiding the direct participation of biological degradation via chemical and extracellular transport through efflux of P-glyco proteins. At last, polymeric-NPs increased the bioavailability of drugs into the brain. Polymeric-NPs exhibits the small particle size, it is simply transported transcellularly by olfactory neurons to the brain by other endocytic pathways of neuronal cells to treat Parkinson’s disease [26–28]. Therefore, these nanoparticles can be a better approach in the comparison of in situ nasal-gel.

Poly (lactic-co-glycolic acid)-Nanoparticles (PLGA-NPs) with optimal size, shape, and ligand attached particular surface have been broadly used for drug targeting of intranasal to brain [29]. For various drug delivery systems, polyvinyl alcohol nanoparticles (PVA-NPs) were also used due to its higher solubility, greater permeability, and increase compatibility of mixture in addition to different shapes and flexibility related to rheological properties [30]. PVA having a very good mucoadhesive property due to which most of the researcher nowadays are focusing PVA as it have enhanced usage in protein and adsorption of metal field [31]. Advantages of polyvinyl alcohol are drug delivery systems delayed release due to mucoadhesive nature; therefore enhancing the drug absorption with sustained release of drug [31–33]. The mucoadhesive nature of PVA lowers the burst release of drug due to entrapment of drug inside the core of polymer/PVA-NPs which further enhances the permeation. PVA-NPs retained due to the attraction between the negatively charged membrane and positively charged PVA [31–33].

Accordingly, we designed to prepare PLGA-NPs for increased brain targeting of THQ. The main aim is to increase THQ bioavailability in brain following an intranasal (i.n.) delivery, to attain a maximum drug therapeutic level in CNS while avoiding the unwanted systemic exposure of drug and reducing the dose requirement for therapeutic advantage. THQ-loaded-NPs will be developed and characterized in order to evaluate their appropriateness for nose-to-brain drug delivery. Current study evaluates the importance of THQ-S and THQ-loaded-PLGA-NPs reaching to all regions of brain and blood along with evaluation of pharmacokinetic parameters (C_max_, AUC_0–t_, t_1/2_, K_el_ etc.) of THQ-S and THQ-loaded-PLGA-NPs. Direct nose-to-brain transport and %DTE (brain targeting efficiency) will be measured after intranasal and intravenous delivery (THQ-loaded-PLGA-NPs, THQ-S).

On the basis of above mentioned detailed conceptualization on beneficiary effect of Thymoquinone in epilepsy by integrating modern scientific methods for drug delivery with their traditional way of clinical use and understand how latest technologies can be applied to authenticate their ethnopractice use.

The concepts of nanotechnology (nanometric formulations) have been used for the selected phytoconstituents, thymoquinone delivered by intranasal route to get greater delivery system for the control of seizure with decreased the dose in the epileptic animal models.

Another most important issue related with THQ-quantification in the different matrixes i.e. lungs, brain as well as plasma. On the basis of previous literature survey various methods have been reported for THQ-analysis in the commercial product and in different geographical plant extracts however individual analysis for THQ in rats brain, lungs as well as plasma is still lacking [34, 35]. This shows the urge for development of a simple, specific and highly sensitive chromatographic method in order to estimate the THQ concentration at nanogram level in the plasma, lungs as well as brain tissue.

Up to the best of our knowledge, current study is a first time study of its kind in order to develop and validate a bioanalytical method for THQ encapsulated in PLGA-NPs via UHPLC. The developed method showed wide application and more efficiency in terms of high sensitivity and low retention time, for successful bioanalytical investigation in plasma as well as brain pharmacokinetics. In addition, current method offers an extra advantage of THQ quantification in the plasma, lungs, and brain tissue homogenate over the CC-range (100.00–2500.00 ng/mL) with a LOD 40.0 ng/mL. The authors have developed a novel-bioanalytical method with retention and run time i.e. 0.463 min and 2.0 min, respectively. The validated-method has another advantage to bioanalyse the more number of samples in a very less time.

## Result and discussion

### Characterization of optimized-THQ-PLGA-NPS

#### Measurement of particle size, PDI and Zeta potential

**Fig. 1:**
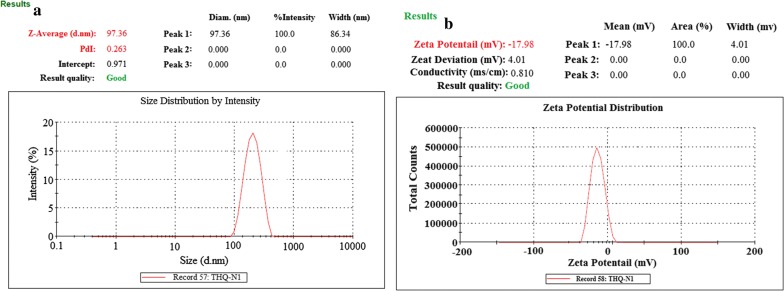
Dynamic light scattering techniques for determining the particle size distribution of THQ-loaded-PLGA-NPs particle size, (**a**) and zeta potential (**b**)

Characterization was performed on the basis of optimized parameters of nanoformulation such as particle size (mean), PDI, and zeta potential as shown in Table 1. All the particle size observed narrow range variability with mean size (97.36 ± 2.01 to 411.96 ± 3.69 nm), dispersion (PDI, 0.263 ± 0.004 to 0.509 ± 0.006), and zeta potential (− 17.98 ± 1.09 to − 31.79 ± 1.59). An increase in PLGA-content followed by increasing the particle size THQ-N1 (100 mg) 97.36 ± 2.01 nm to THQ-N1 (500 mg) 411.96 ± 3.69 nm was observed. It is also reported in the literature, PLGA concentration were increased followed by increase in particle size of NPs [28, 36]. Particles were dispersed narrowly with monodisperse-NPs in Fig. 1 and Table 1.

**Table 1:** Composition of Thymoquinone (THQ)-loaded-PLGA-nanoparticles

Formulation code	PLGA (mg)	DCM (mL)	THQ (mg)	PVA (%)	Mean particle size (nm) ± SD	Polydispersity index (PDI) ± SD	Zeta potential	Entrapment efficiency (EE %) ± SD	Drug loading (DL %) ± SD	Process yield (%) ± SD
THQ-N1	100	8	10	1	97.36 ± 2.01	0.263 ± 0.004	− 17.98 ± 1.09	82.49 ± 2.38	5.09 ± 0.13	88.94 ± 3.48
THQ-N2	200	8	10	1	196.48 ± 2.37	0.371 ± 0.005	− 21.31 ± 1.13	73.16 ± 2.16	5.10 ± 0.11	86.42 ± 3.08
THQ-N3	300	8	10	1	297.16 ± 2.71	0.389 ± 0.005	− 25.46 ± 1.30	70.94 ± 3.11	5.76 ± 0.12	83.017 ± 4.01
THQ-N4	400	8	10	1	372.49 ± 3.23	0.410 ± 0.006	− 28.61 ± 1.41	70.69 ± 3.21	6.33 ± 0.21	77.12 ± 3.14
THQ-N5	500	8	10	1	411.96 ± 3.69	0.509 ± 0.006	− 31.79 ± 1.59	68.75 ± 3.37	6.94 ± 0.48	72.03 ± 4.59

#### %EE and % DL determination

%EE of all THQ-loaded-PLGA-NPs is tabulated in the Table 1. Concentrations of PLGA-polymer were observed with little influence on the entrapment of drug amount in NPs. On the basis of their results, nanoformulae THQ-N1 and THQ-N2 indicated the maximum entrapment efficiencies among all the prepared nanoformulations, having various PLGA-concentrations with their values equal to 88.94 ± 3.48% and 86.42 ± 3.08%, respectively.

Additionally, polymeric effect on %DL for THQ-loaded-PLGA-NPs was also identified as presented in Table 1. Percentage of drug loading was also observed a dependent parameter on the used quantity of polymer ratio i.e. an increase in DL from 5.09 ± 0.13 to 6.94 ± 0.48% was observed for THQ-N1 and THQ-N5, respectively. On the basis of results observation, THQ-PLGA-NPs were given a significantly greater percentage of DL and EE in the comparison of other nanoparticles which is the major cause of PVA-presence.

#### Morphology of particles by scanning electron microscopy

**Fig. 2:**
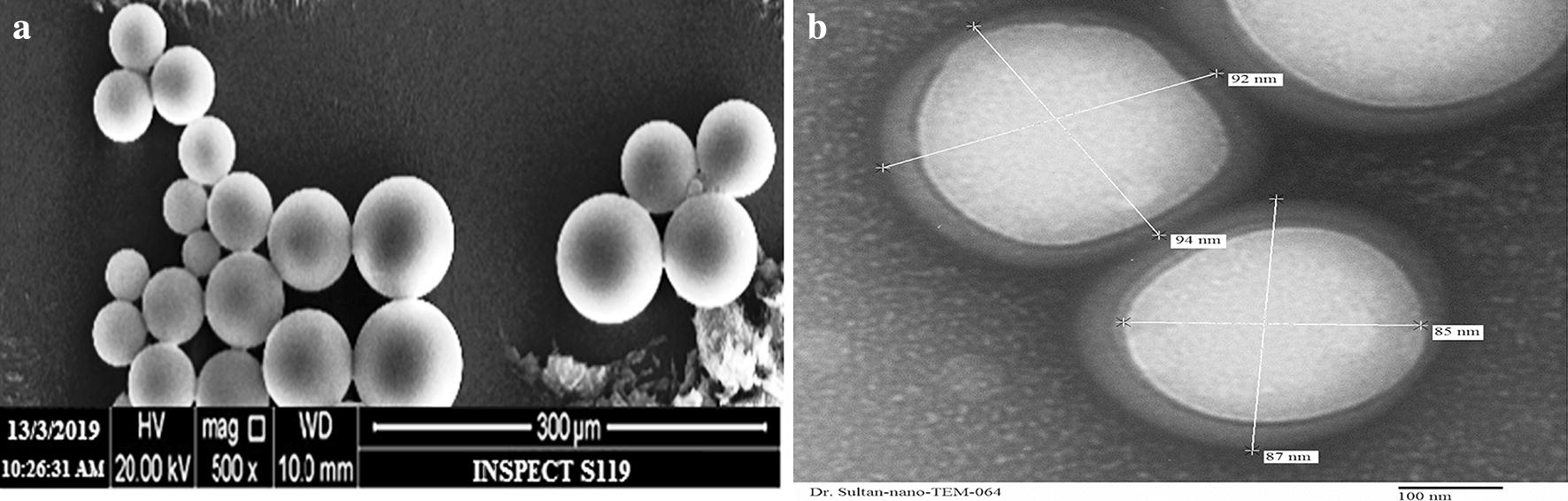
Scanning electron microscopy (SEM) (**a**) and transmission electron microscopy (TEM) (**b**) images of THQ-PLGA-NPs

THQ-loaded-PLGA-NPs (THQ-N1) particles morphology is presented in Fig. 2a. Particles surface was observed with round smooth and fine structure. Aggregation of particles may present due to leftovers of PVA, it has been removed by the 2–3 washing of the particles with Milli-Q-Water. There is expectation of another reason for the aggregation of particles may be at the time lyophillization. We placed the particles on the metallic plating at the time of characterization of SEM. “Additional hypothesis; bonding between the particles may be due to high-energy centrifugal force were applied at the time of isolation of the particles from the dispersion. It can be forced for aggregation of the particles. Finally, it forms the agglomerates between the particles”. At the end we observed equally distribution of THQ-PLGA-NPs and it is clearly appeared spherical shaped having smooth surfaces of the nanoparticles.

#### Analysis of particle size by TEM

Spherical shaped nanoparticles were shown by TEM (Fig. 2b). TEM microscopic-viewfinder has divided into four quadrants and analysed the particle size range (97.36 ± 2.01 nm). All the determinations were found to be approximately same to each other.

#### Differential scanning calorimetry (DSC)

**Fig. 3:**
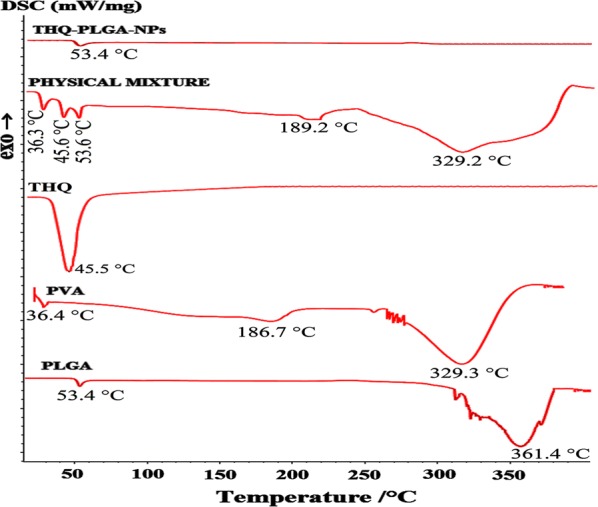
DSC thermograms of pure THQ, PLGA, PV, physical mixtures of THQ, PLGA, PVA; and freeze-dried THQ-PLGA-NPs

The DSC thermograms of THQ, PLGA, PVA, physical mixture of (THQ, PLGA, PVA), and THQ-encapsulated-PLGA-NPs, respectively, are shown in Fig. 3. An experimental study showed a sharp and well-defined endothermic peak at ∼ 45.5 °C equivalent to the melting point of THQ followed by an endothermic broad band. Similarly, the physical mixture of THQ, PLGA, and PVA showed the characteristic peaks of all these. But THQ-sharp peak was absent in THQ-loaded-PLGA-NPs. This indicates THQ completely entrapped in the THQ-loaded-PLGA-NPs. A broad peak of PLGA and PLGA-NPs were shown in-between 45 to 55 °C (Fig. 3) [28, 36, 37].

#### Analysis by ATR based FT-IR

**Fig. 4:**
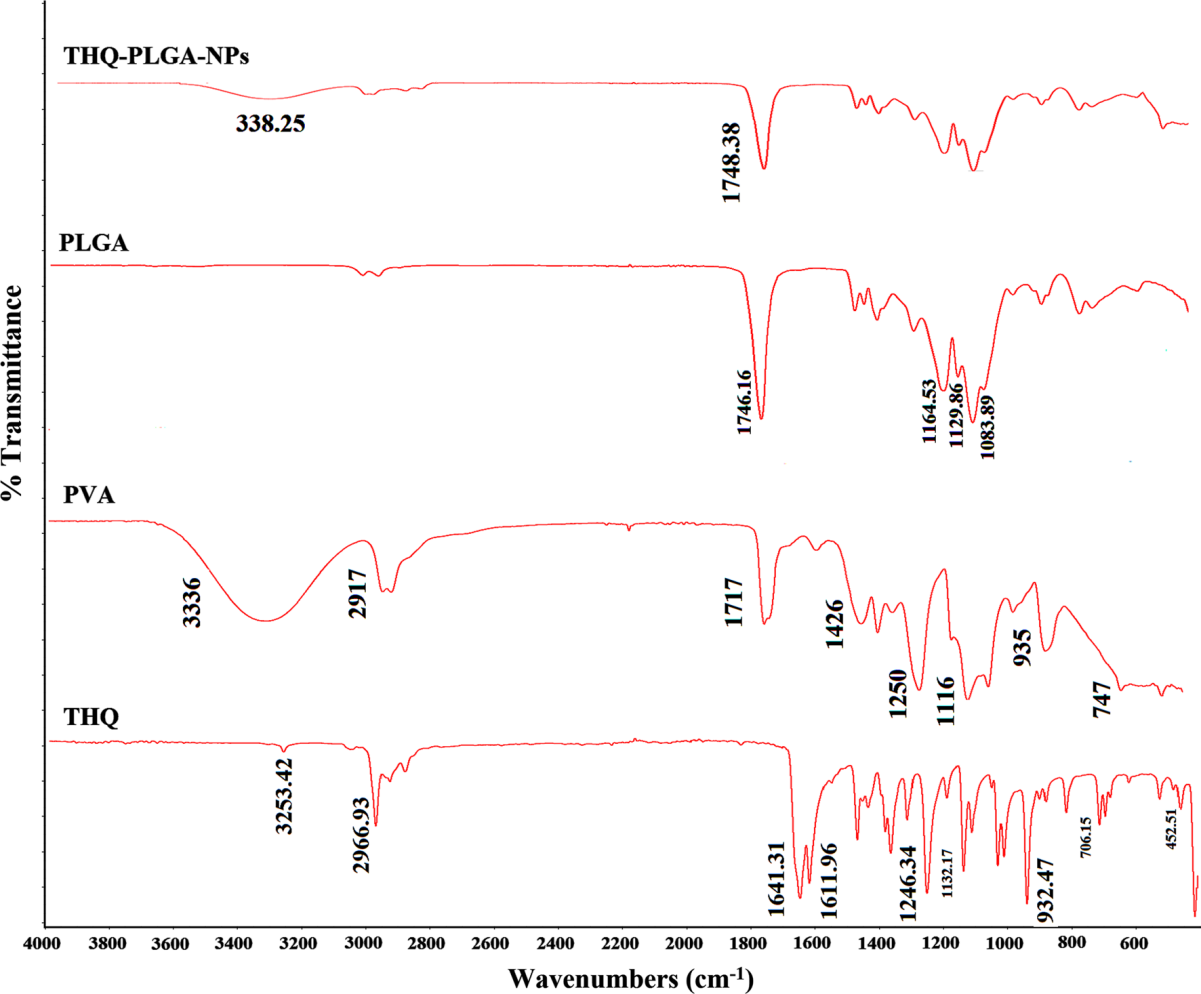
Different FT-IR spectra with an ATR attachment of THQ, PVA, PLGA-NPs, and freeze-dried THQ-PLGA-NPs

PLGA-NPs, THQ, THQ-PLGA-NPs, and PVA were characterized by FT-IR spectrophotometer which had been presented in Fig. 4. THQ, THQ-PLGA-NPs, PVA, and PLGA-NPs were determined through ATR based FT-IR spectroscopy. Nanomaterial surfaces were characterized by the ATR-based-FT-IR spectroscopy which is a multipurpose tool that can be utilized in the both way qualitatively or quantitatively [34, 38, 39]. PLGA-NPs showed their characteristic peaks on 1746.16 cm^−1^ which is corresponds to the carbonyl or –C=O stretching. THQ showed their characteristic peaks at 2964.31 cm^−1^, 1645.13, 1603.67,1356, 1305, 1247.21, 1130, 1019,931, 776 cm^−1^. PVA showed characteristics peaks 3336, 2917, 1717, 1426, 1250, 1116 cm^−1^. THQ-PLGA-NPs achieved all the characteristic peaks of PLGA-NPs. But, THQ-characteristic peak inside the THQ-PLGA-NPs was not found. This is due to ATR-FTIR spectroscopy analysed the nanomaterial surfaces. It is clear indication THQ was maximum encapsulated in the core of THQ-loaded-PLGA-NPs. Although, the little amount of THQ may be present on the surface of nanoparticles. THQ-amount was insignificant to be detected in comparison of THQ-loaded-PLGA-NPs. Finally, it is clearly indicated that there was no possible chemical reactions between the THQ and any one of the nanoformulation ingredients [34, 38, 39]. Furthermore, it was more confirmed that THQ was presented maximum inside the core of the NPs. This is also an ignorance of the isomerization produced through light to facilitate stability and biological activities of THQ.

#### In vitro drug release study

**Fig. 5:**
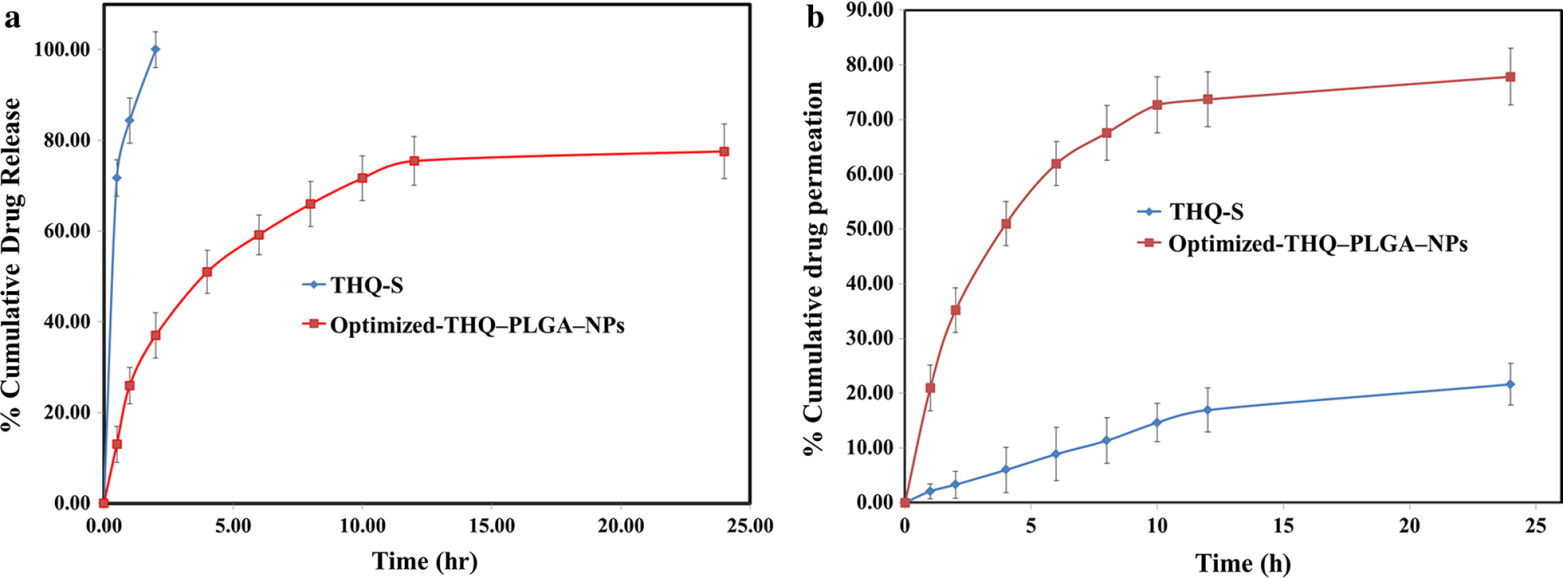
**a** In vitro release profile of THQ-S, and THQ-loaded-PLGA-NPs performed by using dialysis bag method, revealing sustained release pattern of THQ-PLGA-NPs, (mean ± SD, n = 3). **b** Ex-vivo permeation profiles of developed Rut-CS-NPs as compared to pure rutin through goat nasal mucosa

The release profile of THQ from optimized PLGA-NPs exhibited a sustained release pattern. It was detected that the released THQ primarily exhibited a rapid initial release (burst release) followed by a characteristic slow or sustained-release pattern. The initial fast release of drug could be due to release of THQ from the PLGA-NPs surface, while at a later stage, THQ may be constantly released from the core of NPs as a significance of PLGA hydration and swelling [28, 36, 37]. THQ-release study presented a highest release of 77.58 ± 3.84% with sustained release phenomenon from THQ-PLGA-NPs after 24 h on other side comparison of 100% release without showing sustained form with THQ-S (Fig. 5).

#### Nasal mucosa based ex vivo permeation studies

THQ-PLGA-NPs had showed higher permeation than pure THQ-drug -solution (Fig. 5b). Optimized THQ-PLGA-NPs was found significantly (p < 0.001) permeated in comparison of THQ-S. The highest permeation was seen > 77.84% in 24.00 h whereas THQ-S was 21.64% only (Fig. 5b).

#### UHPLC-PDA based bioanalytical method development and validation

**Fig. 6:**
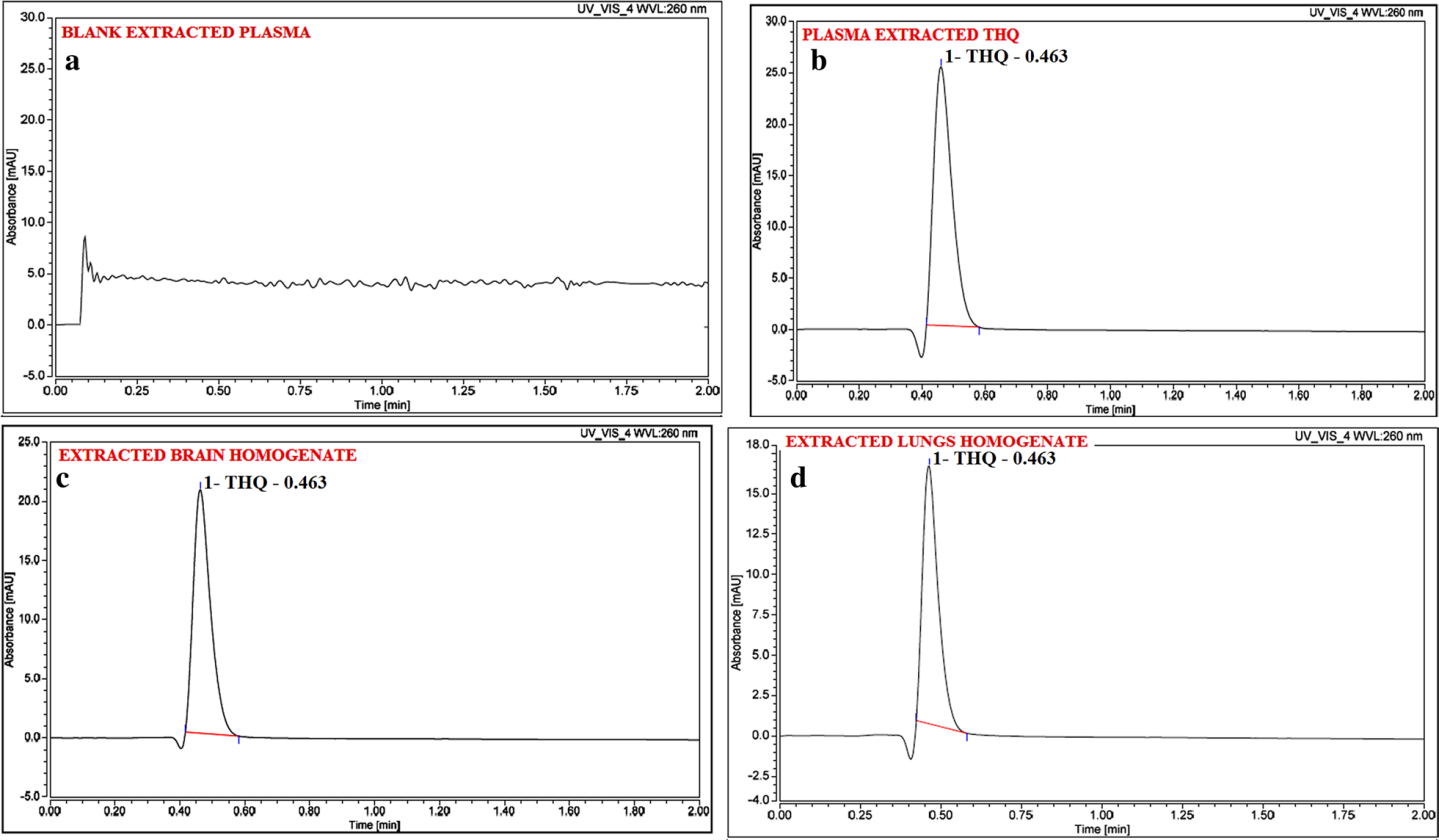
Typical chromatograms of **a** Blank extracted plasma, **b** plasma extracted thymoquinone, **c** brain homogenate extracted thymoquinone, **d** lungs homogenate. Extracted thymoquinone after spiking with Wistar rat-brain homogenate through PDA mode

**Fig. 7:**
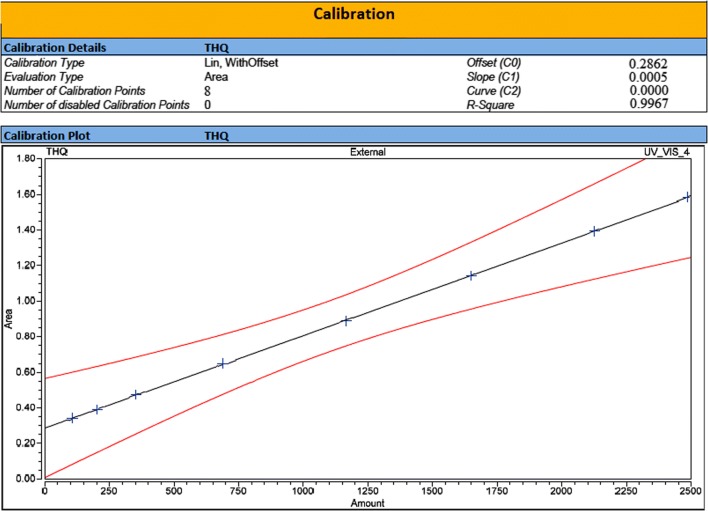
Calibration standard peaks at different calibration standards 100.0, 200.0, 350.0, 680.0, 1160.0, 1650.0, 2125.0, and 2500.0 ng/mL with their calibration curve graph (100.0–2500.0 ng/mL)

All the typical chromatograms were presented in Fig. 6 which have clearly mentioned as in [A] Extracted plasma as a blank, [B] THQ Extracted from Plasma, [C] THQ extracted from brain homogenate, and [D] THQ extracted from lungs homogenate. THQ mean recovery (n; 6) of plasma and brain homogenates were > 83.0%. THQ-bioanalytical method was established and shown to be linear (r^2^ > 0.9967) in the plasma, lungs, and brain homogenate over the CC-range (100.00–2500.00 ng/mL) (Fig. 7). Extracted blank plasma chromatogram represents and determined THQ which clearly proved the selectivity of the method. Table 2 shown precision and accuracy data of Inter-day and intra-day. In a summarized form, the data obtained for %CV of intra-batch and inter-batch to the entire QC levels of THQ-range were 0.96 to 1.91 and 1.02 to 1.86 for all biomatrices i.e. plasma, BH, and LH. Accuracy for intra-batch and inter-batch were found in the limits 95.50 to 99.26 and 94.68 to 99.02% for all biomatrices i.e. plasma, BH, and LH to all QC levels of THQ (Table 2). All storage conditions like bench-top stability, long-term stability, freeze-thaw stability, and post-processing stability showed method stability of THQ in the Table 3 [39–41].

**Table 2:** Validation: precision and accuracy data for thymoquinone in different biomatrixes

Biomatrix	Quality controls samples	Theoretical concentration (ng mL^−1^) or (ng g^−1^)	Intra-batch precision			Inter-batch precision			Recovery^c^ (%)
			Observed concentration (ng mL^−1^) or (ng g^−1^) ± S.D.	Accuracy^a^ (%)	Precision^b^ (%C.V.)	Observed concentration (ng mL^−1^) or (ng g^−1^) ± S.D.	Accuracy^a^ (%)	Precision^b^ (%C.V.)	
Brain Homogenate	LOQQC	101.00	97.79 ± 1.38	96.82	1.41	96.63 ± 1.27	95.67	1.31	85.34
	LQC	290.00	276.94 ± 3.64	95.50	1.31	274.56 ± 3.11	94.68	1.13	86.47
	MQC	1000.00	979.64 ± 11.46	97.96	1.17	974.43 ± 11.17	97.44	1.15	87.61
	HQC	2000.00	1985.22 ± 27.11	99.26	1.37	1982.42 ± 26.64	99.12	1.34	88.23
Lungs Homogenate	LOQQC	101.00	97.68 ± 0.934	96.71	0.96	98.28 ± 1.14	97.31	1.16	83.14
	LQC	290.00	282.46 ± 3.75	97.40	1.33	279.99 ± 3.64	96.55	1.30	88.18
	MQC	1000.00	976.49 ± 15.47	97.65	1.58	974.94 ± 14.99	97.49	1.54	87.23
	HQC	2000.00	1981.44 ± 25.14	99.07	1.27	1980.47 ± 24.34	99.02	1.23	87.43
Plasma	LOQQC	101.00	99.64 ± 1.16	98.65	1.16	99.31 ± 1.01	98.33	1.02	88.32
	LQC	290.00	281.45 ± 3.93	97.05	1.40	279.64 ± 3.81	96.43	1.36	86.93
	MQC	1000.00	976.42 ± 18.69	97.64	1.91	972.25 ± 18.14	97.23	1.86	87.39
	HQC	2000.00	1976.48 ± 26.18	98.82	1.32	1974.14 ± 25.22	98.71	1.28	87.24

**Table 3:** Validation: stability data for thymoquinone in different biomatrixes

Exposure condition	LQC(290.0 ng/mL or ng g^−1^)			MQC(1000.0 ng/mL or ng g^−1^)			HQC (2000.0 ng/mL or ng g^−1^)		
	Brain homogenate	Lungs homogenate	Plasma	Brain homogenate	Lungs homogenate	Plasma	Brain homogenate	Lungs homogenate	Plasma
Long term stability; recovery (ng) after storage (− 80 °C)									
Previous day	288.9 ± 0.93	288.6 ± 0.50	288.6 ± 0.54	994.6 ± 13.36	992.6 ± 14.6	993.4 ± 17.4	1993.6 ± 26.4	1992.6 ± 27.8	1989.1 ± 27.5
30th day	281.2 ± 0.91 (97.33%)	287.1 ± 0.45 (99.48%)	284.1 ± 0.91 (98.44%)	982.4 ± 15.6 (98.77%)	988.6 ± 15.4 (99.60%)	984.6 ± 17.1 (99.11%)	1981.4 ± 24.1 (99.39%)	1988.2 ± 26.1 (99.78%)	1970.4 ± 24.6 (99.06%)
Freeze-thaw stress; recovery (ng) after freeze-thaw cycles (− 80 °C to 25 °C)									
Pre-cycle	287.9 ± 0.67	289.3 ± 0.81	289.1 ± 0.64	993.7 ± 15.6	993.4 ± 14.6	991.5 ± 16.4	1990.4 ± 27.0	1991.8 ± 27.3	1990.6 ± 26.4
First cycle	284.1 ± 0.90 (98.68%)	286.6 ± 0.86 (99.07%)	288.2 ± 0.74 (99.69%)	985.4 ± 13.9 (99.16%)	988.4 ± 2.46 (99.50%)	987.9 ± 17.5 (99.64%)	1981.7 ± 26.9 (99.56%)	1988.4 ± 26.4 (99.83%)	1981.4 ± 27.4 (99.54%)
Second cycle	282.3 ± 0.92 (98.05%)	283.4 ± 0.94 (97.96%)	286.47 ± 0.66 (99.09%)	979.4 ± 14.6 (98.56)	985.7 ± 14.2 (99.22%)	986.4 ± 16.3 (99.49%)	1975.4 ± 23.8 (99.25%)	1981.5 ± 25.8 (99.48%)	1975.4 ± 26.1 (99.24%)
Third cycle	280.3 ± 0.93 (97.36%)	280.1 ± 0.93 (96.82%)	285.94 ± 0.91 (98.91%)	975.6 ± 15.4 (98.18%)	982.4 ± 14.5 (98.89%)	979.9 ± 17.1 (98.83%)	1971.1 ± 24.7 (99.03%)	1974.2 ± 26.7 (99.12%)	1970.9 ± 25.4 (99.01%)
Bench top stability; recovery (ng) at room temperature (25 °C)									
0 h	288.4 ± 0.91	287.8 ± 0.91	288.47 ± 0.71	992.8 ± 14.6	991.4 ± 14.7	989.9 ± 16.8	1992.4 ± 25.4	1989.1 ± 28.1	1991.5 ± 26.7
24 h	286.3 ± 0.78 (99.27%)	282.3 ± 0.81 (98.09%)	286.44 ± 0.84 (99.30%)	986.4 ± 15.0 (99.36%)	984.6 ± 13.5 (99.31%)	981.4 ± 15.8 (99.14%)	1981.5 ± 27.1 (99.45%)	1977.6 ± 27.5 (99.42%)	1979.1 ± 27.4 (99.38%)
Post processing stability; recovery (ng) after storage in auto sampler (4 °C)									
0 h	289.3 ± 0.34	288.6 ± 0.81	289.21 ± 0.64	993.4 ± 14.9	993.4 ± 14.5	988.7 ± 17.6	1991.4 ± 26.3	1988.9 ± 28.3	1988.3 ± 23.4
4 h	287.5 ± 0.62 (99.38%)	281.94 ± 0.49 (97.69%)	287.3 ± 0.84 (99.34%)	989.6 ± 13.6 (99.62%)	986.6 ± 13.9 (99.32%)	981.2 ± 18.0 (99.24%)	1983.1 ± 25.7 (99.58%)	1971.5 ± 26.4 (99.13%)	1970.1 ± 22.9 (99.08%)

Values (Mean ± SD) are derived from six replicates. Figures in parenthesis represent analyte concentration (%) relative to time zero. Theoretical contents; LQC: 290.0 ng mL^−1^; MQC: 1000.0 ng mL^−1^; and HQC: 2000.0 ng mL^−1^

#### Biodistribution with pharmacokinetics (%DTP and %DTE)

**Fig. 8:**
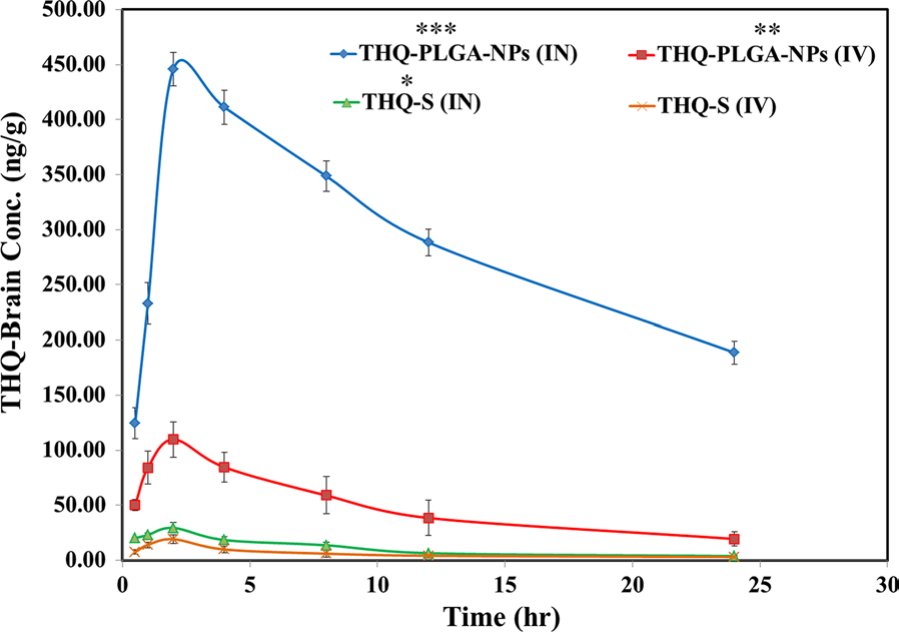
Pharmacokinetic Profile of Thymoquinone (THQ) concentration in brain at different time intervals after administration of developed THQ-loaded-PLGA-NPs compared with pure THQ. Significance was determined as ***p < 0.001, **p < 0.01, *p < 0.05, when compared with pure THQ (IV) solution

Table 4, Fig. 8 displays the comparative PK-parameters (T_max_, C_max_, K_e_, t_1/2_, and AUC_0–t_) of THQ-PLGA-NPs with THQ-pure-solution after administration by i.n. and i.v. AUC_0–t_ of THQ-PLGA-NPs were found significantly higher when it compared to THQ-S in all parts (lungs, brain, and plasma). Drug concentration of brain was found to be lower in i.v. administration as compared to intranasal administration. Table 4 showed the brain/plasma ratio. Table 5, Fig. 8 exhibited the %DTE, %DTP, and AUC_i.n_/AUC_i.v._; which was clearly indicated the bioavailability is enhanced by i.n. route for optimized THQ-PLGA-NPs.

**Table 4:** Pharmacokinetic parameters of THQ-PLGA-NPs after i.n. and i.v. administration to rats at the dose of 10 mg kg^−1^ in brain, lungs and plasma (n = 6, mean ± SD)

Formulation administration	Samples	Cmax (ng/mL g)	Tmax	t_1/2_ (h)	Ke (h^−1^)	AUC_0–t_ (ng min/mL g)
THQ-S (i.n.)	Brain	29.46 ± 1.67	2.00	8.22 ± 0.75	0.0843 ± 0.009	251.08 ± 15.97
	Lungs	14.89 ± 0.96	2.00	20.61 ± 1.08	0.0336 ± 0.0021	186.30 ± 20.10
	Plasma	12.89 ± 0.64	0.5	5.61 ± 0.57	0.1237 ± 0.0021	53.07 ± 1.56
THQ-S (i.v.)	Brain	19.17 ± 0.88	2.00	10.43 ± 0.46	0.0664 ± 0.0011	145.68 ± 10.09
	Lungs	12.09 ± 0.76	2.00	11.95 ± 1.00	0.0580 ± 0.0011	130.47 ± 9.95
	Plasma	491.01 ± 11.19	1.00	7.74 ± 0.24	0.0895 ± 0.0031	3832.45 ± 121.08
THQ-PLGA-NPs (i.n.)	Brain	445.94 ± 8.67	2.00	87.98 ± 12.46	0.0079 ± 0.00009	6942.67 ± 109.89
	Lungs	102.01 ± 7.68	2.00	15.29 ± 1.87	0.0453 ± 0.0006	776.63 ± 28.89
	Plasma	23.01 ± 1.87	2.00	13.07 ± 2.67	0.0530 ± 0.0021	260.64 ± 28.06
THQ-PLGA-NPs (i.v.)	Brain	109.84 ± 9.01	2.00	11.29 ± 1.67	0.0614 ± 0.0035	1154.31 ± 78.01
	Lungs	91.01 ± 4.66	2.00	23.12 ± 2.09	0.0300 ± 0.0048	800.12 ± 34.11
	Plasma	453.93 ± 16.01	1.00	9.21 ± 0.96	0.0753 ± 0.0014	3675.41 ± 98.95
TQ (i.n.)	Brain/plasma	2.29	4.00	1.47	0.68	4.73
TQ (i.v.)	Brain/plasma	0.03	2.00	1.35	0.74	0.04
THQ-PLGA-NPs (i.n.)	Brain/plasma	19.38	1.00	6.73	0.15	26.64
THQ-PLGA-NPs (i.v.)	Brain/plasma	0.24	1.00	1.23	0.82	0.32

**Table 5:** Drug targeting efficiency and direct nose-to-brain transport following intranasal administration of different formulations

Formulations	Drug targeting efficiency (%DTE)^a^	Direct nose-to-brain transport (%DTP)^a^	Comparative bioavailability^a^ (AUC_i.n._/AUC_i.v._); (%)	
			Blood	Brain
THQ-S	7633.33 ± 91.78	47.01 ± 4.98	1.38 ± 0.12	6.55 ± 0.83
THQ-PLGA-NPs	8075.00 ± 113.05	89.89 ± 9.38	5.07 ± 0.67	98.24 ± 6.89

^a^Parameters are derived using mean ± SEM values of 6 different estimations

Release of drug is mostly by various mechanisms including desorption from the surface of PLGA-NPs, diffusion from biodegradable polymer particles, polymeric network and degradation erosion, polymeric re-adsorption by pores [28]. THQ release observed from THQ-PLGA-NPs which showed first quick and burst release (36.99% in 2.00 h), followed by a slow and almost sustained release up to 24.0 h. THQ showed burst release initially, due to a release from the THQ-PLGA-NPs outer surface. It proceeds constant extended release of THQ presenting the swelling of polymer and hydration [37]. It is rapid dissolution (36.99%, 2 h) of THQ-S presented fast burst release (Fig. 5a).

Brain bioavailability is enhanced due to the burst release of THQ from THQ-PLGA-NPs with PVA creating it effective in the treatment of epilepsy via THQ. Higuchi model showed linear r^2^ = 0.987 for in vitro kinetic release. This is due to THQ released through both swelling and diffusion in controlled way of the studies [28]. Nasal goat mucosa was used for ex vivo*/*in vitro nasal permeation for THQ-PLGA-NPs. The results were observed maximum permeation as THQ to enhanced permeation effect of PLGA with PVA (with mucoadhesive property) [31–33, 36, 37].

THQ-biodistribution study was performed with the help of UHPLC-PDA followed by %DTE and %DTP. Hence, drug uptake is dependent on the principal routes as reported in literature (Ahmad et al.) i.e.; i) THQ absorbed by systemic pathway to blood circulation to crossed BBB to reach into the CNS; ii) lymphatic-pathway, and iii) epithelium of nasal mucosa to brain via trigeminal nerves or olfactory evading the BBB [27, 41].

Finally it proved that %DTP of THQ-PLGA-NPs values (i.n.) when compare to THQ-S (i.n.) enhances from 47.01 ± 4.98 to 89.89 ± 9.38% proving an enhance in THQ-PLGA-NPs uptake into the brain in comparison of THQ-S (p < 0.01) following i.n. route of administration has presented here the advantage of THQ-PLGA-NPs. The values of relative bioavailability were showed with 6.55 ± 0.83% for THQ-S to 98.24 ± 6.89% for THQ-PLGA-NPs when we compared it. Highest bioavailability was observed from i.n. to brain which is a direct involvement of this pathway [27, 41]. Likewise, comparative bioavailability of THQ-PLGA-NPs and THQ-S is enhanced into the brain when we applied statistics for i.n. application. It is clear indication of nose to brain targeting as reported as the previous research study [27, 28, 42]. Simply on the basis of statistical analysis, Pharmacokinetic Profile of Thymoquinone (THQ) Concentration in brain at different time intervals after administration of developed THQ-loaded-PLGA-NPs compared with pure THQ. THQ-loaded-PLGA-NPs observed the highly significant value by intranasal (***p < 0.001), THQ-loaded-PLGA-NPs (IV) (**p < 0.01), THQ-S (IN) (*p < 0.05), when all compared with pure THQ (IV) solution.

### Pharmacodynamic study for epilepsy

#### Effect of THQ on ICES by THQ-PLGA-NPs

**Fig. 9:**
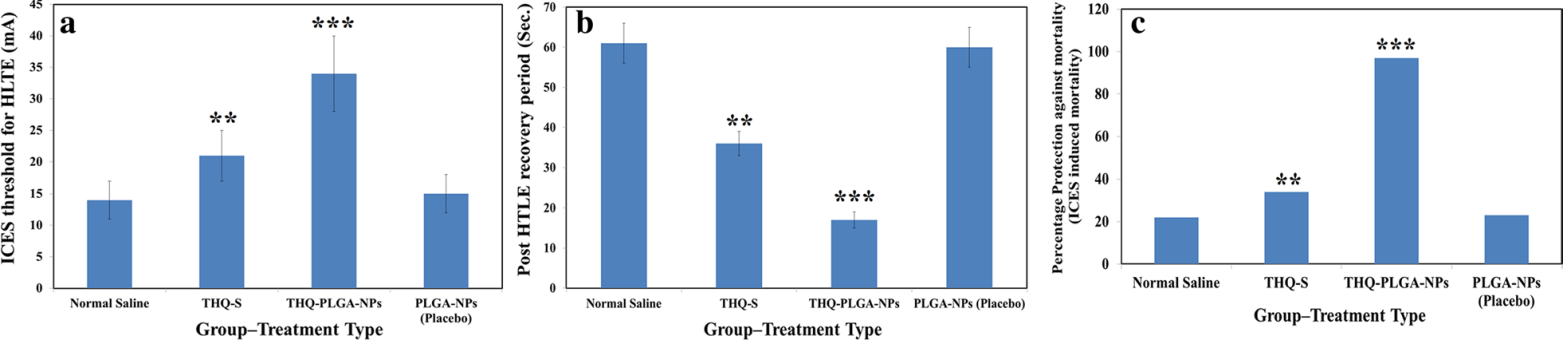
**a** ICES induced threshold for HLTE, **b** ICES induced post HLTE recovery time, and **c** %protective effect of THQ-loaded-PLGA-NPs against ICES induced mortality in rats

THQ-PLGA-NPs (10 mg/kg-body-wt) were highly significant (P < 0.01) in the ICES observation when it compared to THQ-S i.e. a greater significant protection (P < 0.001) was seen as compared to control-group (Fig. 9).

In the proposed study, we found a greater anticonvulsant action (ICES-models) for THQ-PLGA-NPs when i.n. route administered as compared to another routes. Though comparatively, we observed the similar effect with THQ (a very low dose of i.e. 10 mg/kg body wt) administered as i.n. THQ-loaded-PLGA-NPs having very fewer side effects when the drug reached directly to the brain [9, 18–20]. On the basis of above observation, we conclude; a nanoformulation of THQ reached maximum quantity via i.n. route to the brain. It directly reached to the brain through i.n. route as compared to other routes administration. Here, i.n. route has showed more effectiveness than pother routes, it supports reported literatures whereas same as improved anticonvulsant effect of drugs (Lamotrigine, Clonazepam, Diazepam, and Midazolam) in nanoformulation or mucoadhesive nanoformulation was observed [43–45]. The activity enhanced for nanoformulation based THQ-PLGA-NPs is distributed because of ability of nano-carrier across BBB.

## Conclusion

Current study aimed to enhance the bioavailability and sustain release of THQ via nanoformulation (THQ-PLGA-NPs). Different concentrations of PLGA were applied to prepare five different THQ-loaded-nanoformulations (THQ-N1 to THQ-N5). THQ-N1 was observed as an optimized nanoformulation as per optimum parameters studied. THQ-N1 revealed a controlled released pattern (in vitro) for HQ, as confirmed by pharmacokinetic evaluation (in vivo). SEM and TEM evaluated the nano size of globular and smooth surface of the nanoparticles while ex vivo and DSC studies exhibited an enhanced solubility for THQ with an entrapment in the core of NPs. THQ-PLGA-NPs effectively treated epilepsy with small doses followed by crossing the BBB. UHPLC-PDA-based-bioanalytical method was successfully developed, and validated. It was applied successfully for the biodistribution and pharmacokinetic studies of optimized THQ-PLGA-NPs. THQ-PLGA-NPs was applied and treated successfully on ICES model for epilepsy. THQ-PLGA-NPs showed an innovative, safe with effective brain-targeted delivery system in the treatment of epilepsy. Therefore, it is finally concluded that the optimized THQ-PLGA-NPs benefits from the nanosized and promise better therapeutic efficacy.

## Materials and methods/experimental

Thymoquinone (purity 99.98%) was purchased from Frinton Laboratories, 4204 Sylon Boulevard, Hainesport, New Jersey, 08036, USA. Poly (vinyl alcohol, MW 25000) from Polysciences Inc, 400 Valley Road and PLGA and DCM from Sigma‑Aldrich Corporation (St. Louis, MO, USA) were purchased. HPLC‑grade methanol, acetonitrile, ammonium formate, ammonium acetate, and formic acid were purchased from Sigma‑Aldrich Corporation (St. Louis, MO, USA). Milli‑Q‑Water was used in the whole analysis. All the other chemicals used that were of analytical grade were obtained from different commercial sources.

### Preparation of nanoparticles

THQ-PLGA-NPs were prepared and optimized through previously used method i.e. emulsion solvent evaporation method with slight modification [28, 37]. In a summarized form, drug (10.00 mg) was dissolved in PVA solution (400 µL, 1.0% w/v, and pH 3.0 adjusted) and PLGA (100.00 mg) was dissolved in DCM (8.0 mL). Emulsification of polymeric solution was sonicated (over an ice bath) in drug solution for 1.5 min (duty cycles (40%), 25 W, Sonopuls, Bandelin, Germany). Previously prepared w/o primary emulsion was added drop by drop up to 8.0 mL of aqueous phase (external, 1.0% w/v PVA) under sonication (25% amplitude, over an ice bath, 2.0 min). Finally, resultant dispersion preparation was exposed under mild magnetic stirring (400.00 rpm) for evaporation of solvent at the room temperature. The nano-suspension, after solvent evaporation, was centrifuged (with 18,000 rpm for 20.0 min.) and the pellet obtained was washed, lyophilized (24.0 h at − 50 °C, 0.015 mbar pressures) to attain a free flowing and simply dispersible lyophilized nanoparticles (Lab Conco., LPYH, Lock 6, USA freeze dryer).

### Particle size, PDI (polydispersity index), and Zeta Potential

Measurement of particle size is the most important parameter. Smaller particle size increase the surface area for enhanced absorption of the drug [25, 28, 37]. Particle size, polydispersity index, and zeta potential of optimized-NPs was measured with the help of Dynamic light scattering (Malvern-Zetasizer, Nano-Zetasizer, UK). The nano-formulations were diluted before size analysis, temperature was maintained (25 °C) and scattering angle was fixed (90°) [25, 28, 37].

### Scanning electron microscopy (SEM)

The shape of particles was determined by scanning electron microscopy (SEM) ((FEI, INSPECT S50, Check Republic)). The proposed method was adopted from Ahmad et al. [25, 28, 37].

### Transmission electron microscopy (TEM)

The globule size of optimized nanoparticles was determined via transmission electron microscopy (TEM) (FEI, MORGAGNE.68, Check Republic). The proposed method was adopted from Ahmad et al. [25, 28, 37].

### Loading capacity (%LC), encapsulation efficiency (%EE), and %process yield of prepared and optimized-nanoparticles

%EE and %LC of NPs was determined by the ultracentrifugation (at 15,000 rpm; 30 min at 4 °C). Previously UHPLC-PDA method was used to evaluate and validate the free quantity of THQ in supernatant [25, 28, 37]. Following a triplicate measurements, the following equation was used to calculate LC (%) and EE (%) for developed and optimized-nanoparticles [25, 28, 37]:

$$ \begin{aligned} {\text{EE }}\left( \% \right) \, = \frac{{{\text{Total Quantity of THQ }}{-}{\text{ Free Quantity of THQ}}}}{\text{Total Quantity of THQ}} \times 100 \hfill \\ {\text{LC }}\left( \% \right) = \frac{{{\text{Total Quantity of THQ }}{-}{\text{ Free Quantity of THQ}}}}{\text{Weight of Nanoparticles}} \times 100 \hfill \\ \end{aligned} $$

Process Yield (%) as calculated as;

$$ {\text{Process Yield }}\left( \% \right) \, = \frac{{{\text{W}}_{ 1} \left( {\text{Weight of Dried Nanoparticles}} \right)}}{{{\text{W}}_{ 2} \left( {\text{Total Dried Weight of Starting Materials}} \right)}} \times 100 $$

### DSC (differential scanning calorimetry) study

DSC 214 Polyma (NETZSCH‑Wittelsbacherstraße 42, 95100 Selb, Germany) was used to determine the DSC of Pure-THQ, Polymer (PLGA), Physical Mixture of optimized Polymer (PLGA) + THQ, and freeze-dried-THQ-loaded-optimized Polymer (PLGA)-NPs. Sample (10 mg) was kept inside of standard aluminium pan, -crimped, and -heated (15 °C to 400 °C) at a rate of 10°K/min followed by continuous supply of nitrogen [25, 28, 37].

### FT-IR with ATR

Functional groups of the compounds with their chemical structure and composition were characterized through FT‑IR i.e. ATR (NICOLET iS50 FT-IR; Thermo Fisher Scientific, 5225 Verona Road, Madison, WI 53711, USA). IR-spectra of THQ, PVA, PLGA-NPs, and THQ-loaded-PLGA-NPs were determined by an attenuated total reflectance (ATR, wavenumber 4000–400 cm^−1)^. Pure THQ, THQ-PLGA-NPs, and PLGA-NPs (Placebo) were directly analysed without any special preparation.

### In vitro drug release

In-vitro release of drug from optimized-NPs was performed via a pre-treated dialysis membrane (pore size: 2.4 nm-molecular-weight cut-off ∼ 12–14 kD) [25, 28, 37]. Initially the release medium (phosphate buffer:ethanol of 7:3; pH 7.4 = 100 mL). The temperature was maintained (37 ± 1 °C) up to 6 h at 100 rpm, with the help of a stirrer. Finally, dialysis bag properly check for any leak and we will place inside THQ-NPs (containing 0.50 mg THQ). For the THQ-release study from the NPs selected predetermines selected time points (i.e. 30, 60, 120, 240, 360, 480, 720, 1440 min). At every time point, we have withdrawn the test samples (1 mL). All the withdrawn samples filtered by syringe filter (0.45 μm) first after that THQ quantity analysed through the in house developed and validated UHPLC-method reported in this manuscript [25].

### Nasal mucosa based ex vivo permeation studies

Fresh nasal tissues were taken out from goats nasal cavities, arranged from a local slaughterhouse. Tissue cells (A fix area) decided to permeate the drug (0.785 cm^2^; Logan Instrument Corporation, Piscataway, NJ). Phosphate buffer saline (20 mL PBS; pH 7.4, at 37 °C) was added to the receptor chamber while Pure-THQ-Solution and freeze-dried-THQ-loaded-optimized Polymeric-PLGA-NPs (~ 10.0 mg of THQ) kept in the donor chamber (21.5 to 2 mL) after preincubation-time (20 min in each case). A 0.500 mL samples (on predetermined time intervals) were withdrawn from receptor chamber and filtered via a membrane filter for the analysis. The quantity of permeated THQ was analysed through an in house developed and validated UHPLC-method reported in this manuscript [25].

### In vivo study

For In vivo pharmacodynamics or pharmacokinetics studies, a proper ethical approval was sorted from Animal ethical approval Committee, Imam Abdulrahman Bin Faisal University (Dammam-Saudi Arabia). All Albino rats were received from the Animal House, Imam Abdulrahman Bin Faisal University (Dammam-Saudi Arabia). Albino rats (weight; 180–200 g) were grouped (5 to 10 in each cage) and maintained natural light followed by dark cycle with free reachable to food and water (temperature 20.0 to 30.0 °C, humidity; 50.0 to 55.0%). All animals were kept to laboratory prescribed conditions. Research activity was started during the light cycle in wake up condition using freely moving animals.

For Anesthesia and euthanasia: Ketamine (40–90 mg/kg body weight) have been used as analgesia at the time of the surgical procedure (i.e. cardiac puncture for blood collection).

If still needed at the time of surgical procedure, the researchers have given Isoflurane (Dose: 2% inhaled form in desiccator).

At time of development of MCAO Model (i.e. cerebral ischemia) we have given anesthesia to develop a model:

Dose: Ketamine: 40–90 mg/kg + Xylazine 5–10 mg/kg

Route: Intraperitoneal (IP)

### Bioanalytical development of method and their validation for Thymoquinone by Ultra-High-Performance liquid chromatography-Photodiode Array Detector (UHPLC-PDA)

For bio analytical method development and validation, Thermo Scientific™ Vanquish™ UHPLC system (Thermo Scientific, Germany) made up of a binary solvent delivery system alongwith photodiode array detector (Chromeleon (c) Dionex Version 7.2.8.10783, Germany) was used to perform UHPLC. For chromatographic separation the tools used with specifications were as; Pinnacle DB Cyanom (1.9 µm; 30 mm × 2.1 mm), degassed mobile phase of HPLC-grade solvent i.e. Methanol:Water (80:20 v/v) with isocratic elution, flow rate (0.300 mL min^−1^) as well as injection volume of 5.0 µl as injected at every run. The total run time was 2.00 min and also retention time was 0.463 min., with software Chromeleon (c) Dionex Version 7.2.8.10783.

We were followed US-FDA guidelines at the time of development of method and all parameters of validation for greatest-fit relationship of concentration-detector-response with a regression equation (1/x^2^) were used [40].

### Biodistribution, pharmacokinetics (PK), brain drug-targeting-potential, and drug-targeting efficiency

Biodistribution and Pharmacokinetics (PK) were performed on 8 rats in of 4 groups (Total 4 group) (Total Rats: 32 = 4 × 8). THQ-S and THQ-loaded-optimized Polymeric (PLGA)-NPs (Dose: 10 mg/Kg-body-weight) was given to rats separately intranasal (i.n.) as well as intravenous (i.v.) followed by previously chosen time points (30, 60, 120, 480, 720 min and also at 24 h). The blood collected at selected these time points and bioanalyse the THQ concentration as per the newly in house developed reported method in this article mentioned research study [25, 28, 37]. Finally, we had evaluated the all the pharmacokinetics parameters [C_max_, t_1/2_ and AUC_(0–t)_] in the separated plasma.

Group I: THQ-S (intranasal)

Group II: THQ-S (intravenous)

Group III: THQ-loaded-optimized polymeric (PLGA)-NPs (intranasal)

Group IV: THQ-loaded-optimized polymeric (PLGA)-NPs (intravenous)

All the observation data acquired from biodistribution bioanalysis was utilized to evaluated for Drug Targeting Efficiency (%DTE) and Brain Drug-Targeting-Potential (%DTP) through mentioned below formulae: [23, 25, 28, 37].

$$ {\text{DTE\%}} = \left[ {\frac{{\left( {{\text{AUC}}_{\text{brain}} /{\text{AUC}}_{\text{blood}} } \right)_{{{\text{i}} \cdot {\text{n}} \cdot }} }}{{\left( {{\text{AUC}}_{\text{brain}} /{\text{AUC}}_{\text{blood}} } \right)_{{{\text{i}} \cdot {\text{v}} \cdot }} }}} \right] \times 100 $$

$$ {\text{DTP\%}} = \left[ {\frac{{B_{{{\text{i}} \cdot {\text{n}} \cdot }} - B_{x} }}{{B_{{{\text{i}} \cdot {\text{n}} \cdot }} }}} \right] \times 100 $$

where, ${\text{B}}_{\text{x}} = \frac{{{\text{B}}_{\text{iv}} }}{{{\text{P}}_{\text{iv}} }} \times {\text{P}}_{\text{in}}$; B_x_ = AUC for brain fraction; B_iv_ and P_iv_, B_iv_ and P_iv_ = brain and blood AUC_0to24_ (after i.n. and i.v. administration).

### Pharmacodynamic activity

#### Experimental procedures for increasing-current electroshock seizure (ICES) test [21, 46, 47]

Ahmad et al. [21], Marwah et al. [46] and Kitano et al. [47] method was used to evaluate the anticonvulsant effects of test samples through ICES test. Six rats were used in each and every group. In brief, 2 mA, electroshock was given initially on one train of pulse (20 Hz square wave for 0.2 s); was delivered to each separate rat by ear electrodes. If the 2 mA for 0.2 s applied in the ICES test is not suitable to develop the intensity of seizure threshold current (STC). After that the Intensity of electroshock was increased linearly as 2 mA/2 s and started to observe the point suitable for tonic hind limb extension (HLE). The point was noted for current intensity of seizure threshold current (STC). Furthermore, absence of tonic HLE up to a current (30 mA), cessation of electroshock for final result with cut off current was given to analysis.

### Statistical analysis

All outcomes were evaluated and represented as Mean ± SEM (Standard Error of Mean). Student’s t-test was used for the unpaired observations calculation and their difference with the help of ANOVA i.e. p-value.

## Data Availability

The datasets generated during and/or analysed during the current study are available from the corresponding author on reasonable request.

## Abbreviations

UHPLC-PDA: Ultra high performance liquid chromatography with Photodiode Array Detector
THQ: Thymoquinone
NPs: Nanoparticles
PPT: Protein precipitation
LLE: Liquid–liquid extraction
SPE: Solid-phase extraction
LOD: Lower limit of detection
LOQ: Lower limit of quantitation
LLOQ: Lower limit of quantification
LLOQ QC: Lower limit of quantification for quality control
LQC: Low quality control
MQC: Middle quality control
HQC: High quality control
PLGA: Poly(lactic-co-glycolic acid)
PDI: Polydispersity index
ZP: Zeta potential
PVA: Polyvinyl alcohol
SEM: Scanning electron microscopy
TEM: Transmission electron microscopy
EE: Entrapment efficiency
LC: Loading capacity
FTIR: Fourier-transform infrared spectroscopy
ATR: Attenuated total reflectance
C_max_: Maximum plasma concentration
K_el_: Elimination rate constant
Tmax: Time to Cmax
t_½_: Half-life
AUC: Area under curve
ICES: Increasing-current electroshock seizure
